# O-FIB: far-field-induced near-field breakdown for direct nanowriting in an atmospheric environment

**DOI:** 10.1038/s41377-020-0275-2

**Published:** 2020-03-16

**Authors:** Zhen-Ze Li, Lei Wang, Hua Fan, Yan-Hao Yu, Hong-Bo Sun, Saulius Juodkazis, Qi-Dai Chen

**Affiliations:** 1 State Key Laboratory of Integrated Optoelectronics, College of Electronic Science and Engineering, Jilin University, Changchun, 130012 China; 2 State Key Laboratory of Precision Measurement Technology and Instruments, Department of Precision Instrument, Tsinghua University, Beijing, 100084 China; 3 Nanotechnology Facility, Swinburne University of Technology, John St., Hawthorn, 3122 Vic Australia

**Keywords:** Laser material processing, Laser-produced plasmas, Nanophotonics and plasmonics, Lithography

## Abstract

Nanoscale surface texturing, drilling, cutting, and spatial sculpturing, which are essential for applications, including thin-film solar cells, photonic chips, antireflection, wettability, and friction drag reduction, require not only high accuracy in material processing, but also the capability of manufacturing in an atmospheric environment. Widely used focused ion beam (FIB) technology offers nanoscale precision, but is limited by the vacuum-working conditions; therefore, it is not applicable to industrial-scale samples such as ship hulls or biomaterials, e.g., cells and tissues. Here, we report an optical far-field-induced near-field breakdown (O-FIB) approach as an optical version of the conventional FIB technique, which allows direct nanowriting in air. The writing is initiated from nanoholes created by femtosecond-laser-induced multiphoton absorption, and its cutting “knife edge” is sharpened by the far-field-regulated enhancement of the optical near field. A spatial resolution of less than 20 nm (*λ*/40, with *λ* being the light wavelength) is readily achieved. O-FIB is empowered by the utilization of simple polarization control of the incident light to steer the nanogroove writing along the designed pattern. The universality of near-field enhancement and localization makes O-FIB applicable to various materials, and enables a large-area printing mode that is superior to conventional FIB processing.

## Nanotechnology: Better writing with light

An optical version of Focused Ion Beam technology (FIB) allows nanoscale “writing” such as surface texturing, drilling and sculpting of materials to be performed in air, avoiding the need for a vacuum which limits the application of conventional FIB. The “Optical Far-field-Induced near-field Breakdown” (O-FIB) approach has been developed by Hong-Bo Sun of Tsinghua University and colleagues at Jilin University in China and Swinburne University of Technology in Austrilia. It works by creating nanoholes with a femtosecond laser, which is controlled by sophisticated optical effects. The process can cover larger areas than conventional FIB, and with a spatial resolution below 20 nanometres. The ability to be performed in an open atmosphere offers new possibilities for nanoscale writing. These range from working on industrial scale materials such as ship hulls, down to living tissues and cells.

## Introduction

Lasers are becoming one of the dominant tools in the current manufacturing industry^1–4^. Much effort has been devoted to improving the processing accuracy, and spatial resolutions as low as micrometers have been achieved in laser cutting, welding, marking, and stereolithography in an atmospheric environment^3–6^. The femtosecond laser (fs laser) is a particularly promising approach from this point of view, in addition to its three-dimensional (3D) processing capability^7–12^ and broad-spectrum material usability^13–18^. Sub-diffraction-limited feature sizes at a level of tens of nanometers based on multiphoton absorption^19,20^, thresholding^21^, shrinkage^22,23^, and stimulation emission depletion effect^24,25^, have also been realized in fs-laser-induced photocuring of polymers, which unfortunately are not applicable to solid materials. Optical near-field techniques provide an alternative super-resolution scheme by localizing light fields to nanometer scales with the physical shapes of sharp tips^26^, tiny apertures^27^, nanoparticles^28,29^, and small protrusions^30^. Nevertheless, these approaches often rely on complex movement and alignment systems to maintain precise probe–substrate spacing for practical fabrication/patterning throughput due to the evanescent nature of the near field. An innovative optical patterning technology that permits vacuum-free high-resolution processing comparable to conventional FIB processing is highly desired. Optical far-field-induced near-field breakdown, which we abbreviate as O-FIB, combines the concepts of FIB with the advantages of fs-laser-induced multiphoton excitation and optical near-field superresolution, and enables far-field nanofabrication in the atmosphere that is applicable to almost any solid material. O-FIB demonstrates a new concept of dynamic far-field control of the near-field enhancement and localization at the nanoscale, with the possibility of nanowriting in a manner that is fully determined by the beam’s trajectory and polarization.

## Results

### Nanoholes as seeds for near-field enhancement

**Fig. 1:**
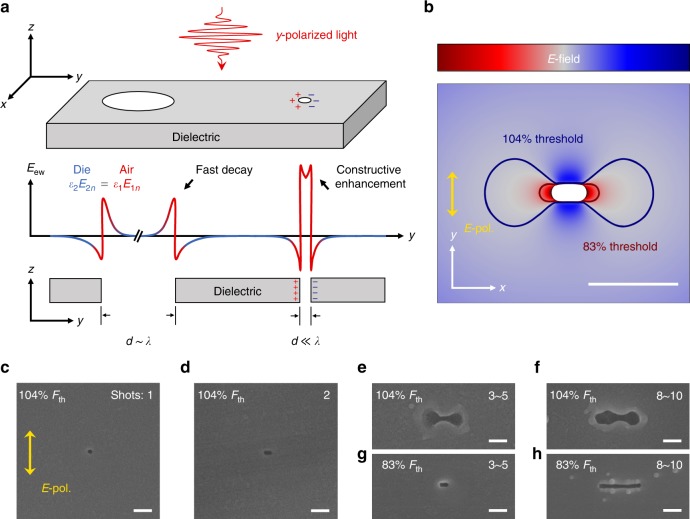
Theory and experimental verification of O-FIB. **a** Schematic plot of the evanescent wave (*E*_ew_) around two nanoholes with different sizes on a dielectric. For the larger nanohole with a diameter comparable to the wavelength, the evanescent fields at each edge of the nanohole are independent and decay rapidly from the boundary. For the smaller nanohole with a deep-subwavelength aperture, the strong interaction between the two boundaries constructively enhances the optical intensity inside the nanohole, and confines the light on a deep-subwavelength scale. The marks “+” and “−” indicate the polarization charges around the nanohole. **b** Simulations of the *E*-field strength distribution for a titanium oxide film with a nanohole shown by the central white-out region (the intensity of the light inside the nanohole is close to maximum, but is not shown for a better visualization). **c**–**h** Direct experimental evidence of the near-field enhancement induced by nanoseeds. With an increase in the pulse number for in situ exposure, the nanohole elongates (**c**–**e**) and finally evolves into a dumbbell crater (**f**) or a uniform groove (**g**, **h**), depending on the laser fluence (104 or 83% of the threshold, *F*_*th*_), as predicted in (**b**). The laser pulse duration is *t*_*p*_ = 150 fs, the wavelength is *λ* = 800 nm, and linear polarization (see the *E*-field orientation marker) is used. Focusing is carried out with an objective lens with a numerical aperture of NA = 0.8, corresponding to a focal spot diameter of *D* = 1.22*λ*/NA *~*1.2 μm. All scale bars are 100 nm

Fs-laser direct nanowriting is initiated with a nanohole as a seed (Fig. 1), which is ablated by few fs-laser pulses on the surface of a solid-state sample via multiphoton absorption^31,32^. Optical near fields are formed at the edges of the nanohole (Fig. 1a) upon irradiation with subsequent laser pulses, where the normal component of the displacement **D** (parallel to the light *E* field, Fig. 1a) is continuous through the dielectric-air boundary (perpendicular to the light propagation direction). The boundary condition requires *ε*_1_*E*_1*n*_ = *ε*_2_*E*_2*n*_, where 1 and 2 denote air and the dielectric, respectively, and *ε* is the permittivity that governs local *E* fields at the nanoscale. Provided that the hole size is sufficiently small (Fig. 1a), the two near fields from the opposite edges will be constructively enhanced, proportional to the square of the refractive index, *n*^2^. The enhancement of the near-field intensity inside the nanohole, *κ* = (*E*_1*n*_/*E*_2*n*_)^2^, is therefore proportional to *n*^4^; hence, for example, *κ* = 3.8 for fused silica, and *κ* = 26.6 for titanium oxide. It is sufficient to carry out nanoablation that is well separated from the surroundings of the nanohole, where a subthreshold intensity does not cause ablation. A similar phenomenon has also been observed in slot waveguides^33–35^, where light is concentrated and guided along the low-index slot.

The nanohole-enhanced optical near field provides the spatial resolution and reduces the light intensity required for laser ablation at the nanoscale. As a proof, Fig. 1c demonstrates a nanohole with a diameter of 20 nm that is ablated by a single fs-laser pulse with a fluence of ~4% above the breakdown threshold (*F*_*th*_) on the surface of titanium oxide. Once the seed is formed, the energy deposition of the second pulse is elongated perpendicular to the *y*-polarized *E* field (Fig. 1d, e), in accordance with the near-field enhancement prediction (Fig. 1b). Upon irradiation with subsequent laser pulses, the nanohole finally evolves into a dumbbell shape (Fig. 1f) or a uniform nanogroove (Fig. 1g, h) for a pulse number of *N* > 8, according to the fluence of the subsequent laser pulses, which provides direct evidence of the near-field enhancement.

### Self-regulation effect and the spatial resolution of patterning

**Fig. 2:**
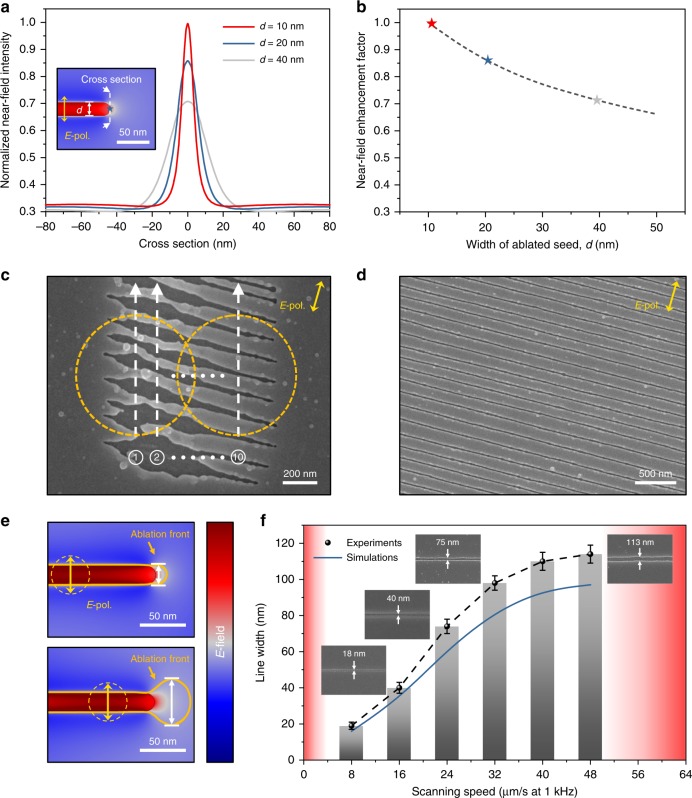
Self-regulation of O-FIB. **a** Normalized near-field intensity profiles along the cross section of the ablated seeds with different widths, *d*. The cross section is indicated by the white dashed line in the inset. **b** Near-field enhancement factor *η* versus the width of the seed *d*; *η* is defined by the normalized near-field intensity at the center of the cross section. **c** Self-regulation of the nanogrooves with a raster (along the *y*-axis) scan after ten subsequent scans are shifted laterally by 100 nm each. **d** Uniform array of grooves after self-regulated nanoablation at the tips of the grooves. **e** Dependence of the ablation front on the focal center (yellow dashed circles) close to the end of the groove. When the focal center is close to the endpoint of the groove, the ablation front exceeding the ablation threshold is wider, resulting in the ablation of a larger groove (orange solid). **f** Experimental and theoretical results of the relation between the intrapulse spacing (controlled via the scanning speed) and the ablated width of the grooves at a fixed pulse energy of *E*_*p*_ = 16.3 nJ. The scanning speed should not be too small or too large since it may result in over-/underexposure compared with the exposure required for the formation of the grooves (red regions)

The size and shape of the nanowriting seed—the nanohole—are not well controlled due to the intrinsic nonlinearity of the multiphoton-absorption-induced nanoexplosion; however, the ablated line widths of the “nanopen” are still perfectly controllable and reproducible. This is attributed to self-regulation, as shown in Fig. 2a, b. The near-field enhancement decreases as the hole size increases, implying that a larger initial seed leads to a weaker near-field hot spot, and finally results in a narrower ablation line with subsequent irradiation and laser beam scanning. Figure 2c, d demonstrates the evolution from an array of randomly shaped seeds (leftmost ends of the grooves, Fig. 2c) to periodic nanogrooves by a raster scan (Fig. 2d). The randomness of the initial seeds gradually evolves into a uniform subwavelength array (see Fig. S8). O-FIB has inherent robustness against the stochastic nature of the initial ablation, and the ultimate spatial resolution for a fixed material and focusing condition is determined by two factors: the laser pulse energy and the scanning speed.

The combination of the two sets of parameters leads to a slightly complicated yet certain and reproducible dependence. Figure 2e, f shows the simulation and experimental results for a moderate scanning speed range (MSR), and that the line resolution is inversely proportional to the speed. This result is counterintuitive but understandable by considering the relative position between the nanoablation front and the location of the focal spot. A faster scan results in a smaller separation between the center of the focus and the ablation front (Fig. 2e), leading to a wider ablated line width. A lower or a higher speed with respect to the MSR results in a worse resolution or isolated ablation pits, respectively. The ideal nanowriting parameter window defined by the exposure dose (influenced by the scan speed, repetition rate, and pulse energy) is generally quite wide, spanning up to one order of magnitude. Within this condition, nanogrooves with uniform widths from 113 ± 5 nm down to 18 ± 3 nm have been realized (Fig. 2f), comparable to those achieved with standard FIB processing.

### Polarization control for free direct nanowriting

**Fig. 3:**
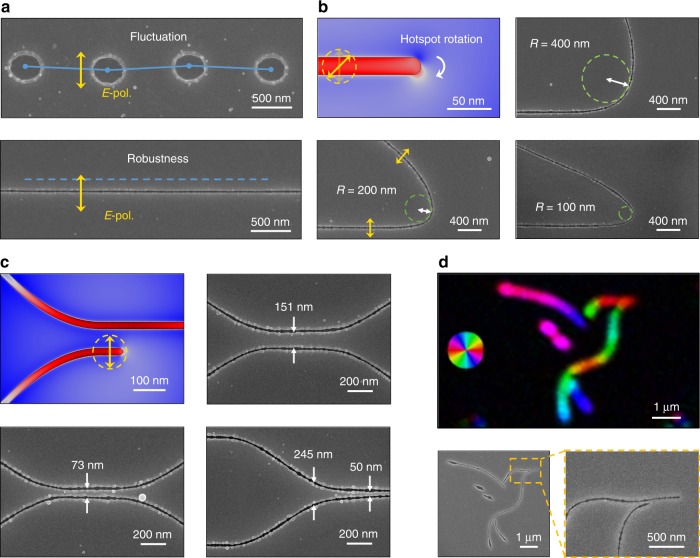
Polarization control of near-field ablation. **a** Robustness of O-FIB against a stage/scanning fluctuation. Upper: the separated craters ablated by a single pulse make the fluctuation of the focus path evident. Lower: a single nanogroove guided by polarization using beam scanning. Due to the strong light confinement inside the nanogroove, the sideway movement of the far-field beam is negligible in the nanoablated line. **b**, **c** Curvature and separation control of O-FIB. The absence of a proximity effect in nanoablation is due to a strong localization of the optical near field. The polarization of the *E* field is shown by yellow markers. The nanogrooves shown in **b**, **c** were recorded with an irradiance ~4% above the ablation threshold. **d** Slow-axis orientation maps (pseudocolor, Abrio) and SEM images of the free-form written nanogrooves. The retardance at a wavelength of 546 nm is 9 nm

O-FIB allows the free writing of arbitrary planar patterns, for which polarization control is critical (Fig. S2). This is a natural requirement, considering that the near-field enhancement always occurs at the two sides of the nanogroove/hole in the polarization plane (Fig. 1a, b and Fig. S4). Curved lines can only be written (nanoablated) freely, as in the case of FIB processing, if the polarization direction is kept perpendicular to the scanning trajectory defined by the predesigned pattern. The capability of O-FIB is demonstrated by the free-form stitchless writing of nanogrooves with a controllable length, separation, and trajectory (Fig. 3). The nanogrooves exhibit significant robustness against a stage/scanning path fluctuation due to the self-regulation effect discussed above (Fig. 3a). A straight line with an alignment error <10 nm is achieved by setting a fixed laser polarization perpendicular to the line trajectory.

Inherently stitchless nanoscale fabrication is realized, which is a strong requirement in lithographic patterning. Curved nanogrooves are achieved by rotating the laser polarization in real time during the laser writing, allowing turning radii as small as 100 nm (Fig. 3b). Because of the strong near-field localization of the pulse energy inside the nanogroove, the crosstalk between adjacent grooves could be effectively eliminated (Fig. 3c and Fig. S7). This property is harnessed to obtain a nanogroove separation that is far smaller than the diffraction limit of ~400 nm (*λ*/2) down to 50 nm or *λ*/16 (Fig. 3c).

The critical role of polarization in O-FIB is not only demonstrated by the well-defined pattern geometry, but also reflected by the optical anisotropy of the nanogrooves. Nanogrooves provide the possibility to engineer birefringence at the nanoscale with programmable azimuth and retardance, from which the variation in the polarization over the course of writing is easily recognizable and can be used for in situ monitoring of nanopatterning. Figure 3d shows the uniform retardance of the free-form patterns, and the orientation of the slow axis in pseudocolor. The retardance of 20-nm-wide nanogrooves is 9 nm measured at a wavelength of 546 nm. The optical image of the nanogrooves is slightly blurred due to the deep-subwavelength character of the imaging, but the birefringence image still provides an additional solid proof of the polarization effect of O-FIB.

### Large-area printing mode

**Fig. 4:**
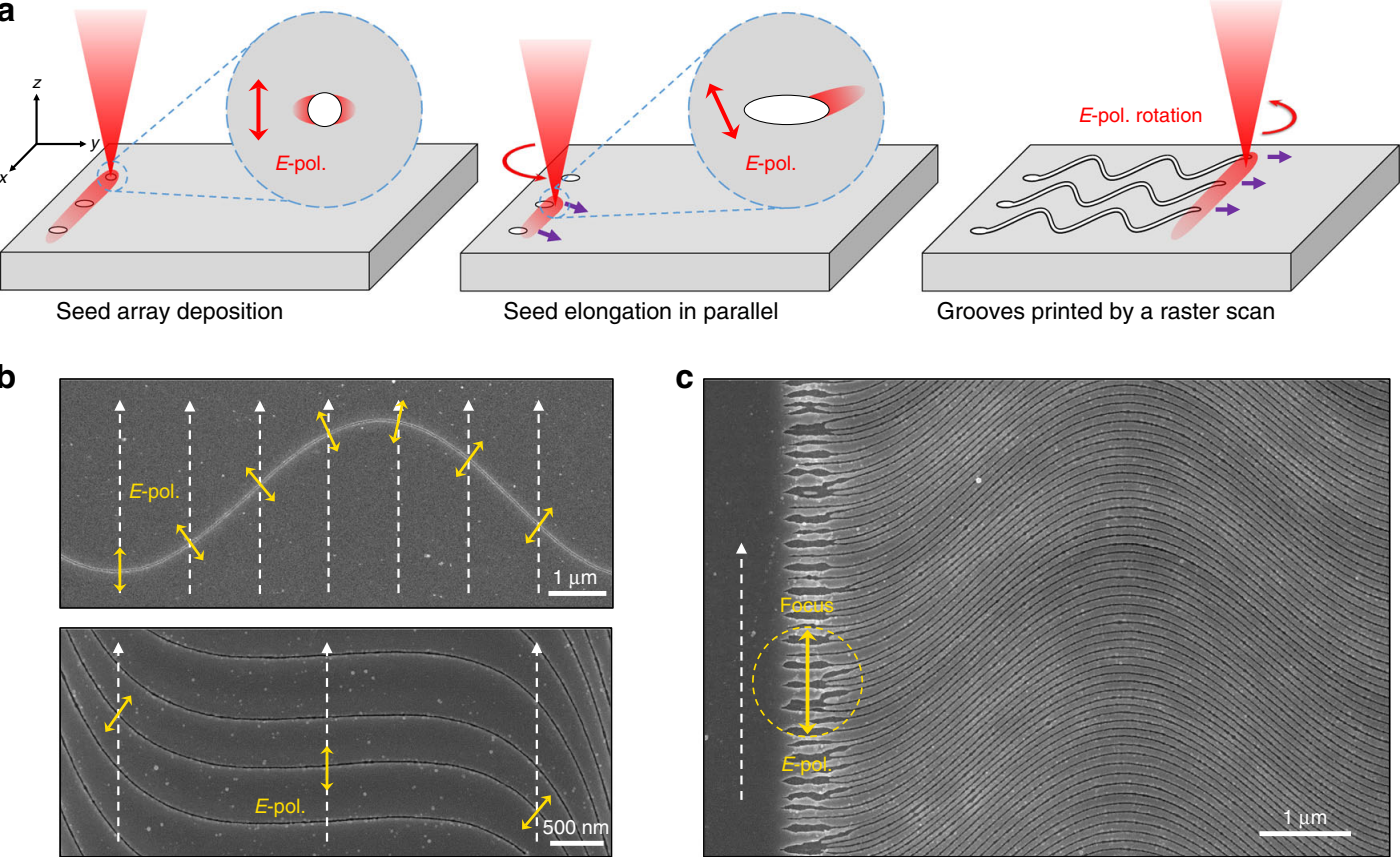
Large-area printing mode. **a** Schematic of the large-area printing mode. **b** A raster scan (vertical) at a speed of 20 μm/s over large areas does not cause damage if there are no nanoholes/grooves for seeding the nanoablation. The growth trajectory of the nanogrooves is always perpendicular to the laser polarization. **c** The initiation and self-organized formation of the nanogrooves over a larger area was carried out at an irradiance 10% above the ablation threshold for a single pulse

The optical near-field enhancement ablation mechanism not only endows O-FIB with a direct writing mode, the sole approach in conventional FIB processing, but also is superior to conventional FIB processing in terms of the capability of large-area printing. An illustration is provided by raster scanning over a large area with predefined nanohole seeds (Fig. 4a and Supplementary Movie 1). The growth direction of each seed is controlled by the polarization at the site during the laser beam scan. This approach allows large-area patterning simultaneously from predefined seeds illuminated by multiple laser foci (Fig. 4b).

A raster scan over a large area at 80% of the ablation threshold irradiance was used to print a pattern of separated nanogrooves from initially preablated nanoholes (on the left side of the pattern). The unstructured areas on the sample are not affected by a light intensity that is 20% lower than the ablation threshold (upper graph of Fig. 4b, Fig. S3), while the pre-existing nanogrooves can still elongate due to the enhancement of the near field at the ablation front (lower graph, Fig. 4b, Fig. S5, and Fig. S6). With an array of initial ablated seeds (Fig. 4c), the ablation sine-wave pattern evolves into a regular grating following line-by-line (vertical) scanning with a step of 100 nm (8% of the focal diameter), without a change in the laser exposure conditions (dose and fluence). The polarization-guided light confinement into the nanogrooves together with the presence of 3–4 grooves per single focal spot favors the light-intensity redistribution and propagation of the nanograting. The efficiency of O-FIB is only limited by the laser repetition rate and the speed of the light polarization modulation. The far-field writing characteristic of O-FIB provides inherent compatibility with digital micromirror devices or spatial light modulators, which can achieve a higher manufacturing speed by manipulating multiple foci in parallel^36,37^.

## Discussion

In summary, we demonstrate a new concept of optical nanofabrication called O-FIB, which enables a sub-20-nm spatial resolution, and can be implemented under atmospheric conditions without the detrimental ion doping that is inevitable in conventional FIB processing. The universal mechanism of the near-field enhancement at nanoholes makes the approach applicable to literally any dielectric or semiconductor material. The approach does not require complicated optical/electric beam manipulation, but relies on simple polarization control during beam steering (or stage scanning). The self-regulation of the nanohole/groove ablation makes this method robust against exposure instability. Due to the inherent stitchless nature of the nanowriting, O-FIB opens a new avenue in the industrial production of nanopatterns and nanodevices by direct writing with ultrafast lasers.

## Methods

A femtosecond (fs) laser (Spitfire, Spectra Physics) was used for ablation. The laser delivers *t*_*p*_ = 150-fs pulses at *λ* = 800 nm, with a repetition rate of 1 kHz. The pulse-to-pulse stability of the laser was ~1.5%. The pulse energy was measured after the objective lens, and the ablation threshold was determined using the probability of ablation for 20 pulses. The ablation pattern was inspected by scanning electron microscopy (JSM-6700F, JEOL). The laser pulses were focused onto the sample with an objective lens, with a numerical aperture of NA = 0.8 (LPL ×80 magnification). The laser beam was scanned by a galvano-mirror XY pair over a field of view of 100 × 100 μm^2^. Films of titanium oxide were deposited over the cover glass using magnetron sputtering. The thickness, roughness, and relative permittivity of the thin film were measured by an ellipsometer (see Fig. S1). Laser ablation was carried out by a single laser pulse, and the threshold-intensity value was determined as *I*_*th*_ = *E*_*th*_ /(*t*_*p*_ A) = 8.5 TW/cm^2^, with a fluence of *F*_*th*_ = 1.3 J/cm^2^ for the pulse energy *E*_*th*_ = 15.6 nJ, a focal spot diameter of *d* = 1.2 μm and the corresponding focal area of A = 1.1 μm^2^. The threshold is determined from the dependence of the ablated pit diameter, *D*_*a*_, on the single-pulse energy, *E*_*p*_ (or *I*_*p*_, *F*_*p*_) by a standard procedure of obtaining a fit by *D*_*a*_^2^ = 2*w*_0_^2^[ln(*E*_*p*_) – ln(*E*_*th*_)], where the intensity is defined as Gaussian, *I*(*r*) = *I*_0_ exp(−2*r*^2^/*w*_0_^2^), and *w*_0_ is the beam waist. The intersection of the linear fit *D*_*a*_^2^ = *f*(ln(*E*_*p*_)) with the *x*-axis (ln(*E*_*p*_)) defines the ablation threshold energy (intensity and fluence), and the slope defines the beam waist, *w*_0_. Numerical modeling of the light-intensity distribution and electronic excitation was carried out with the finite-element method for the experimental conditions using a standard set of material parameters (Table S1). An Abrio (CRi. Inc.) birefringence system was used to measure the retardance and azimuth angle.

## Supplementary information

Supplementary Information

Supplementary Movie 1
