# Supplementary material for: O-FIB: far-field-induced near-field breakdown for direct nanowriting in an atmospheric environment
Source: Light Sci Appl. 2020 Mar 16;9:41. doi: 10.1038/s41377-020-0275-2 (PMC7073331; doi:10.1038/s41377-020-0275-2)
Supplement: Supplementary file 1 — Supplementary Information [file 41377_2020_275_MOESM1_ESM.pdf]

Supplementary Information for "O-FIB: Far-field-induced near-field breakdown for direct nanowriting in an atmospheric environment"

Zhen-Ze Li[1,†], Lei Wang[1,†], Hua Fan[1], Yan-Hao Yu[1], Qi-Dai Chen[1,*], Saulius Juodkazis[2,*] and Hong-Bo Sun[1,3,*]

[1] *State Key Laboratory of Integrated Optoelectronics, College of Electronic Science and Engineering, Jilin University, Changchun 130012, China*

[2] *Nanotechnology facility, Swinburne University of Technology, John st., Hawthorn, 3122 Vic, Australia*

[3] *State Key Laboratory of Precision Measurement Technology and Instruments, Department of Precision Instrument, Tsinghua University, Beijing 100084, China*

[†] *These authors contributed equally to this work.*

[*] *Corresponding authors:*

Hong-Bo Sun: hbsun@tsinghua.edu.cn

Saulius Juodkazis: sjuodkazis@swin.edu.au

Qi-Dai Chen: chenqd@jlu.edu.cn

Contents

1 Necessity to consider the near-field effect in sub-diffraction processing 3

2 Sample selection for mechanism demonstration of O-FIB 4

3 Light E-field at tight focusing 6

4 Interaction of intense laser with material 8

4.1 Nonlinear photo-ionization 8

4.2 Optical response of laser-excited material 10

5 Light confinement inside a nanoslot 13

5.1 Near-field optical intensity around a slot 14

5.2 Stability of the boundary during the laser-excitation 15

5.3 Orientation of the near-field enhancement 16

5.4 Absence of the proximity effect 19

6 O-FIB at high irradiation: self-organization aspects 20

7 References 21

1 Necessity to consider the near-field effect in sub-diffraction processing

In general, the capability of femtosecond laser for sub-diffraction resolultion processing is attributed to the nonlinear threshold effects. In principle, we can obtain arbitrary accuracy as long as the input laser energy is very close to the material breakdown threshold. However, the total energy deposition is not only determined by the shape of focus but also the feedback contributed from material itself. To better illustrate this thought, we approximate the response of the material to the light field as a collection of discrete dipoles [1]. For a normal-incident y-polarized Gaussian beam with amplitude E and waist w, we have the following scattered field for the dipole located at point $\mathbf{r}_0$ [2]:

$$\mathbf{E}_{scat}(\mathbf{r},\mathbf{r_0}) = [(\mathbf{E}_r \sin\theta + \mathbf{E}_\theta \cos\theta)\mathbf{x} + (\mathbf{E}_r \sin\theta + \mathbf{E}_\theta \cos\theta)\mathbf{y}]Ee^{-\frac{{r_0}^2}{2w^2}}, \tag{1}$$

where

$$\begin{aligned} \mathbf{E}_r(\mathbf{r},\mathbf{r_0}) &= 2\beta\cos\theta[\frac{1}{(kR)^2} - \frac{i}{(kR)^3}]\mathrm{e}^{-ikR} \\ \mathbf{E}_\theta(\mathbf{r},\mathbf{r_0}) &= \beta\sin\theta[\frac{i}{kR} + \frac{1}{(kR)^2} - \frac{i}{(kR)^3}]\mathrm{e}^{-ikR}, \end{aligned} \tag{2}$$

with β, the proportional constant; $\mathbf{R}$, vector pointing from $\mathbf{r_0}$ to $\mathbf{r}$; θ, the angle between $\mathbf{R}$ and y-axis; k, the wave vector and i, the imaginary unit. To obtain a deep-subwavelength scale resolution, amplitude E approaches to the breakdown threshold, E_{th}. It implies that, a point belongs to material breakdown region, $\mathbf{R}_b$; must satisfy the near-field condition, $kR_b \ll 1$. Then the rapid-increasing near-field $\sim 1/{R_b}^3$ has a dominant strength compare to the Gaussian component

$\sim E_0$ and the far-field component $\sim 1/R_b$. In this sense, it is the quasi-static near field that totally determines the resolution and ablation morphology when the feature size is reduced to the deep sub-wavelength scale. Once we can regulate and control this near-field, the fs-laser processing will be pushed to its resolution limit.

2 Sample selection for mechanism demonstration of O-FIB

To highlight the significance of the optical near-field during the femtosecond laser processing, the selection of the sample for mechanism demonstration should follows two fundamental instructions:

(a) According to the estimation of intensity enhancement in the main text, $\kappa = (n_2/n_1)^4$; a refractive index difference between sample (n_2) and air (n_1) is required to maintain the near-field enhancement.

(b) To preserve the evidence of near-field ablation, the thermal stability of sample should be good enough to to avoid the melting artifacts and capillary movement on the ablated surface which can alter the initial morphology of the ablated region. Materials with bandgaps E_g falling in multiphoton absoption range ($E_g/\hbar\omega \geq 2$; $\hbar\omega$ is the photon energy, which equals to 1.55 eV at 800 nm wavelength in this study), or thin film with thickness less than 100 nm are the best solutions.

Figure. S1 shows the ellipsometry measurement results of the complex refractive index ($n + ik$) of titania films and glass substrate. Tauc plot was used to determine the bandgap energy of

titamium oxide ~ 3.26 eV. The average thickness of the film is 22.5 ± 2 nm with a roughness of 2.3 nm. The film is dispersionless with a negligible linear absorbance at $\lambda = 800$ nm.

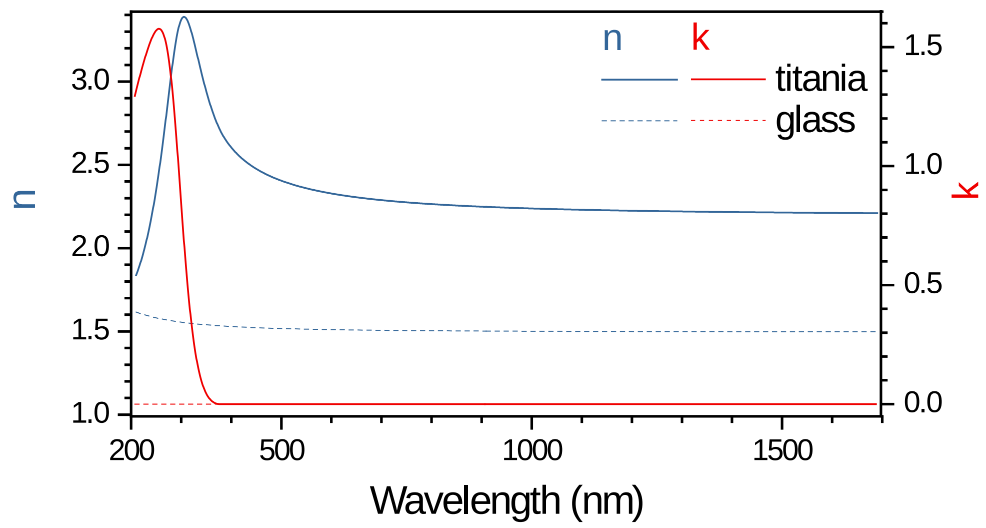

Fig. S1|Optical constants ($n+ik$) of substrate and titanium oxide thin film. Refractive index of substrate and thin film at 800 nm is 1.50 and 2.27, respectively. Also, the imaginary part of the refractive index confirms that the optical bandgap of the titanium oxide thin film should be 3.26 eV.

The illustrated mechanism of the near-field ablation is universal. Similar ablated-seed and self-regulation effect have also been observed on other kinds of thin films, e.g., 20-nm-ZnS (3.7 eV) and 50-nm-Sb_2Te_3 (0.5 eV); or bulk materials, such as $LiNbO_3$ (3.5 eV), YAG (6.5 eV) and fused silica (8.9 eV).

3 Light E-field at tight focusing

The Gaussian beam is regarded as a particular solution of the paraxial Helmholtz equation (following the standard ansatz of definitions for Cartesian coordinates and E-field):

$$\nabla_{\perp}^{2}\mathbf{E}_{\perp} = -2ik\frac{\partial}{\partial z}\mathbf{E}_{\perp}, \tag{3}$$

where $\mathbf{E}_{\perp} = \mathbf{e}_x E_x + \mathbf{e}_y E_y$, $\nabla_{\perp} = \mathbf{e}_x \partial_x + \mathbf{e}_y \partial_y$. This equation stands only when the E-field component along the propagation direction is negligible. However, in our experiments, a high numerical objective $\mathrm{NA} = 0.8$ was utilized to focus the laser pulses onto sample. The strong convergence of light results in the failure of the paraxial approximation. In this case, the first order correction of the E-component could be easily expressed by [3]:

$$E_z = \frac{i}{k}\nabla_{\perp}\cdot\mathbf{E}_{\perp}. \tag{4}$$

A more accurate mathematical description of the tight focused beam is the well-known vectorial integral representation developed by B. Richards and E. Wolf [4]:

$$\mathbf{E}(x,y,z) = -\frac{ik}{2\pi}\int_{\Sigma}\mathbf{T}(n_x,n_y)\mathrm{e}^{-ik(\Phi(n_x,n_y)+n_x x+n_y y+n_z z)}\mathrm{d}\Omega, \tag{5}$$

which can be regarded as a specific form of the plane wave expansion of the electric field near the focus. Here, the surface area integral of $\mathbf{T}(n_x, n_y)$ which is proportional to the E-field strength on the exit pupil was calculated in Cartesian coordinates with $\Phi(n_x, n_y)$ being the aberration function

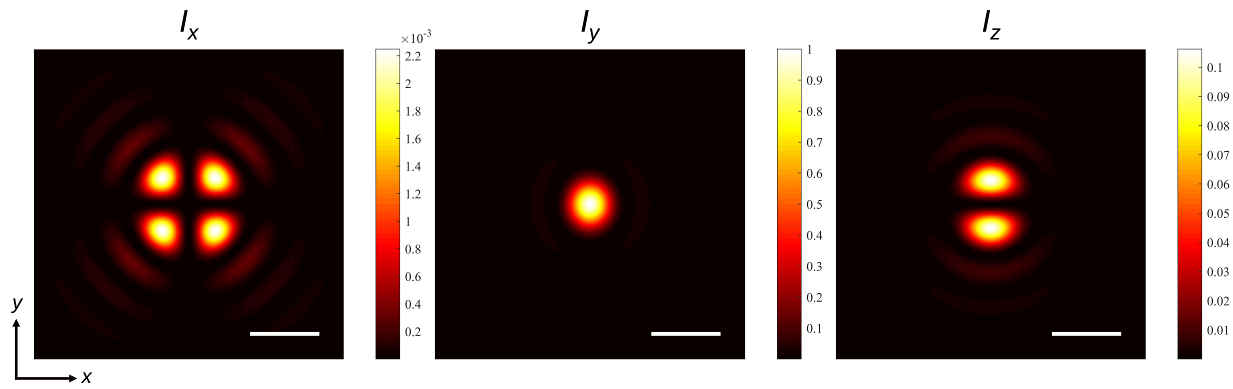

Fig. S2|Intensity of three E-field components at focus. Eqn. 5 was utilized for numerical calculation. All scale bars are 500 nm.

of the system and has been set to zero for simplification; $\mathbf{n} = (n_x, n_y, n_z)$ is the normal vector pointing from a specific surface element on the exit pupil to the focus center and the Ω is the solid angle formed by all the light rays passing through the pupil.

Utilization of high NA lens can cause depolarization effect of E-field due to the strong light convergence. Considering the crucial role of polarization modulation in O-FIB, we need to make sure that the E-field polarization is still well-defined at focus. Here we show the intensity around focus of E_x, E_y and E_z component in x-y plane (Fig. S2). It's clear to see that $I_x \ll I_z < I_y$ maintains an approximate y-polarization around focus.

What has to be emphasized here is that although the mathematical expressions of the tight focused beams are varied, they do not change the basic picture of the light-matter interaction obtained in our simulations. The hole/groove field enhancement originates from the boundary conditions of electromagnetic fields and does not depend on the specific form of the incident beam.

4 Interaction of intense laser with material

The Maxwell's equations were used to simulate the nonlinear coupling between laser light and nano-structures. Set of Maxwell equations in standard notations is following:

$$\begin{aligned}
\nabla \cdot \mathbf{D} &= \rho \\
\nabla \times \mathbf{E} &= -\frac{\partial}{\partial t}\mathbf{B} \\
\nabla \cdot \mathbf{B} &= 0 \\
\nabla \times \mathbf{H} &= \mathbf{J} + \frac{\partial}{\partial t}\mathbf{D}.
\end{aligned} \tag{6}$$

All the responses of nano-structures are reduced to the nonlinearity of the current density, $\mathbf{J}$, and electric displacement, $\mathbf{D}$. More precisely, we want to analyze the transient excited state of the nano-structures and their feedback and contribution to the total electromagnetic field.

4.1 Nonlinear photo-ionization

Hence, we need to consider the nonlinear ionization process first [5]:

$$\Gamma \equiv W_{pi} + W_{av} - W_{re}, \tag{7}$$

where the W_{pi}, W_{av} and W_{re} represent nonlinear photo-ionization, avalanche ionization and recombination rates, respectively. At the initial stage of laser-induced ionization, these dynamical

terms have such forms [6,7]:

$$
\begin{aligned}
W_{pi} &\propto N_0 - N_e \\
W_{av} &\propto N_e(N_0 - N_e) \\
W_{re} &\propto N_e,
\end{aligned}
\tag{8}
$$

where N_e is electron density of the conduction band and N_0 is the density of the available bonds. Then, Eqns. 8 can be written with explicit dependence on the electron density normalized:

$$
\begin{aligned}
W_{pi} &= w_{pi}\frac{N_0 - N_e}{N_0} \\
W_{av} &= w_{av}\frac{N_0 - N_e}{N_0}\frac{N_e}{N_0} \\
W_{re} &= \frac{N_e}{\tau_{re}},
\end{aligned}
\tag{9}
$$

where various concrete forms of rate transitions (model dependent) w_{pi}, w_{av} and τ_{re} (recombination time) are used [5–8]. In any case of specific response, an average electron density can be estimated. We concentrate on how the response of nano-structures at different electron density vary in space and time.

To satisfy the energy conservation, we construct the loss of the electromagnetic field which is related to the nonlinear carrier excitation:

$$
\mathbf{J} \cdot \mathbf{E} = -\nabla \cdot (\mathbf{E} \times \mathbf{H}) - \mathbf{E} \cdot \frac{\partial}{\partial t}\mathbf{D} - \mathbf{H} \cdot \frac{\partial}{\partial t}\mathbf{B}. \tag{10}
$$

A self-consistent way is to set the scalar product of multi-photon current density and the electric field equal to the total energy for interband transitions calculated by the photo-ionization rate [9]:

$$\mathbf{J}_{pi} \cdot \mathbf{E} = \tilde{E}_g W_{pi}, \tag{11}$$

where $\tilde{E}_g \equiv E_g + e^2|\mathbf{E}|^2/4m_0^*\omega^2$ is the effective bandgap, E_g is the optical bandgap and $m_0^* \equiv m_e^* m_h^*/(m_e^* + m_h^*)$ is the reduced effective mass of the electron and hole [8,10,11]. We could also rewrite (Eqn. 11) by defining the nonlinear conductance:

$$\begin{aligned} \mathbf{J}_{pi} &= \sigma_{pi}\mathbf{E} \\ \sigma_{pi} &\equiv \frac{\tilde{E}_g W_{pi}}{|\mathbf{E}|^2}. \end{aligned} \tag{12}$$

Noticing that $W_{pi} \sim o(|\mathbf{E}|^2)$ when $|\mathbf{E}|$ approaches to zero; σ_{pi} will also turn to zero instead of diverging to infinity. Then the nonlinear current terms are well-defined.

4.2 Optical response of laser-excited material

The response of electron-hole plasma at the optical frequency of driving electric field is regarded as a fast small vibration around its equilibrium position [12]:

$$\mathbf{x}(\omega) = \frac{e}{m_0^*}\frac{\mathbf{E}(\omega)}{\omega^2 - i\gamma\omega}, \tag{13}$$

where γ is the damping constant related to momentum/energy loss mechanisms [13]. The amplitude of the electron-hole vibration is so small that it does not make any contribution to the density redistribution. Hence, the diffusion gradient and nonlinear ionization source in the electron density conservation equation are only considered [14]:

$$\frac{\partial}{\partial t}N_e = D_e\nabla^2 N_e + \Gamma, \tag{14}$$

where D_e is the diffusion coefficient. The distribution of plasma density will directly influence the polarization field:

$$\mathbf{D}(t) = \epsilon_0\mathbf{E}(t) + \mathbf{P}(t), \tag{15}$$

with Drude-Lorentz model [12]:

$$\begin{aligned}\mathbf{P}(\omega) &= \sum_{k=1}^{n}\frac{N_i e^2}{m_0^*}\frac{\mathbf{E}(\omega)}{\omega_k^2-\omega^2+i\gamma\omega} + \frac{N_e e^2}{m_0^*}\frac{\mathbf{E}(\omega)}{-\omega^2+i\gamma\omega} \\ &\approx \epsilon_0(\epsilon_r-1)\frac{N_0-N_e}{N_0}\mathbf{E}(\omega) + \frac{N_e e^2}{m_0^*}\frac{\mathbf{E}(\omega)}{-\omega^2+i\gamma\omega},\end{aligned} \tag{16}$$

where $\varepsilon_{0,r}$ are the free space and relative permittivities, respectively. Time response is calculated from that of spectral (frequency):

$$\begin{aligned}\mathbf{P}(t) &= \int \mathbf{P}(\omega)e^{i\omega t}\,\mathrm{d}\omega \\ &\approx \epsilon_0(\epsilon_r-1)\frac{N_0-N_e}{N_0}\mathbf{E}(t) + \mathbf{P}_{e-h}(t),\end{aligned} \tag{17}$$

Table S1|Model parameters for numerical simulations.

Quantity	Symbol	Value/Expression
Refractive index of substrate (800 nm)	n_s	1.50
Refractive index of titania film (800 nm)	n	2.30
Thickness of titania film	d	22.5 nm
Reduced effective mass [15]	m_0^*	$0.8\times9.11\times10^{-31}$ kg
Critical electron density of titania film	N_{cr}	1.39×10^{21} cm^{-3}
Valence electron density of titania film	N_0	1.24×10^{23} cm^{-3}
Damping constant	γ	2.5×10^{14} s^{-1}
Diffusion coefficient [16]	D_e	3×10^{-6} m^2 s^{-1}
Normalized photon-ionization rate	w_{pi}	Reference. [8]
Normalized avalanche ionization rate	w_{av}	Reference. [17]
Electron recombination time	τ_{re}	150 fs

where the contribution of the electron-hole (e-h) plasma for the polarization field should obey the following equation:

$$\frac{\partial^2}{\partial t^2}\mathbf{P}_{e-h} + \gamma\frac{\partial}{\partial t}\mathbf{P}_{e-h} = \frac{N_e e^2}{m_0^*}\mathbf{E}. \tag{18}$$

The above presented formulae was used to calculate e-h generation under used laser exposure conditions plotted in Fig. S8(a).

5 Light confinement inside a nanoslot

Detailed characterisation of the light enhancement and near-field ablation is presented below via numerical and experimental study.

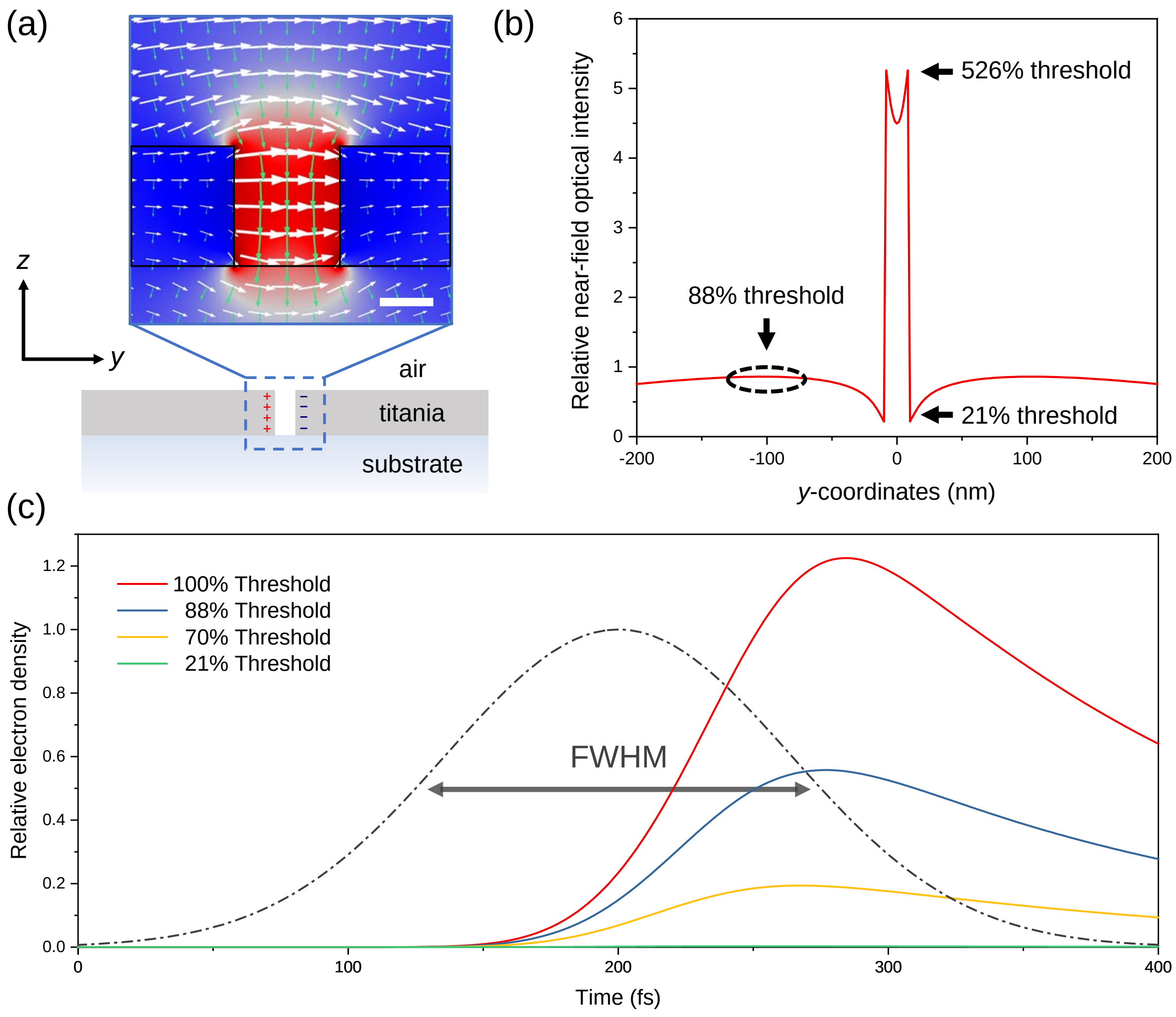

Fig. S3|Stability of the groove's edge under the intense laser pulse irradiation. (a) Distribution of the E-vector (white) and Poynting vector $\mathbf{S} = \mathbf{E} \times \mathbf{H}$ (green) around a 20-nm-width groove on a 22.5-nm-thick titania film (side-view). The E-field strength, as well as the transmission through the air gap (proportional to $\mathbf{S}$), are significantly enhanced. Scale bar is 10 nm. (b) Near-field intensity distribution along y-axis nanogroove. (c) Numerical calculation of the relative electron density (compared to the critical density, $N_{cr} = 1.39{\times}10^{21}$ cm^{-3}) under different excitation intensities. For simplicity, temporal envelope of the pulse (black dashed line) was taken in the form $\exp -[(t - t_0)/\sigma]^2$ with pulse centered at $t_0 = 200$ fs and $\sigma = t_p/2\sqrt{\ln 2}$, where $t_p = 150$ fs.

5.1 Near-field optical intensity around a slot

To simplify description of the spatial light localisation inside the nanohole/groove and also to highlight the physical picture of the confinement effect, we first discuss the light-matter interaction without a nonlinear response. Then, the nonlinear aspect of interaction is taken into account. Finally, we will prove that the linear picture holds due to specific energy deposition around the nanohole/groove.

Consider a 20 nm-width groove irradiated by a y-polarised light at the very threshold of ablation (Fig. S3). According to the Maxwell equations, the electric displacement alongside the titania-air interface should obey the continuous boundary conditions for the displacement $\mathbf{D}$ (no free surface charge) and electric field $\mathbf{E}$ [2]:

$$\begin{aligned} \mathbf{n}\cdot(\mathbf{D}_1-\mathbf{D}_2)&=0 \\ \mathbf{n}\times(\mathbf{E}_1-\mathbf{E}_2)&=0, \end{aligned} \tag{19}$$

which implies for the normal and tangential components:

$$\begin{aligned} \epsilon_1 E_{1n}&=\epsilon_2 E_{2n} \\ E_{1t}&=E_{2t}, \end{aligned} \tag{20}$$

where ϵ_2 represents the linear relative permittivity of dielectric at specific wavelength and $\epsilon_1 = 1$ (air), $\mathbf{n}$ is the vector normal to the interface ($\mathbf{n} \parallel \mathbf{x}$). The light intensity enhancement is defined by

the normal component E_{1n} in the nanoslot:

$$\kappa = (\frac{E_{1n}}{E_{2n}})^2 = \epsilon_2^2 \tag{21}$$

For titanium oxide we have $\epsilon_2 \sim 5.2$, one could find that the electric field is strongly enhanced ($I \propto E_{1n}^2 \approx 26.6$) in a sub-wavelength region by surrounding dielectric materials.

5.2 Stability of the boundary during the laser-excitation

Then, how the nonlinear response of dielectric could change the described intensity distribution? For the time scale of sub-1 ps pulse, one of the main reasons for field-enhancement reduction is a decrease of the refractive index due to electron excitation [10]. The electron density near the boundary can be easily estimated through the relative optical intensity (normalized to threshold) shown in Fig. S3(b). The ablation threshold is considered equivalent to the critical electron density $N_{cr} = \epsilon_0 m^* \omega^2 / e^2$, which corresponds to the plasma density for the resonant absorption of the applied fs-laser pulse [17]. Under the multi-photon ionization approximation, we have $w_{pi} \approx w_{mpi} \propto I^\alpha$, where α is the order of the multi-photon absorption. The optical bandgap of the titanium oxide film is 3.26 eV, which implies a mixture of the two- and three-photon absorption. Then we found the e-h plasma density near the groove's boundary is approximately equal to $(0.04 \sim 0.08) \times N_{cr}$, which only accounts for less than 0.2% of the total valence electrons (accordingly, less than 0.2% change of the linear relative permittivity). A rigorous calculation based on nonlinear ionization rate equation has confirmed this estimation, as shown in Fig. S3(c). Hence, the stability of the groove's

boundary for ablation under the intense laser pulse irradiation can be well understood from a linear picture of the light-matter interaction.

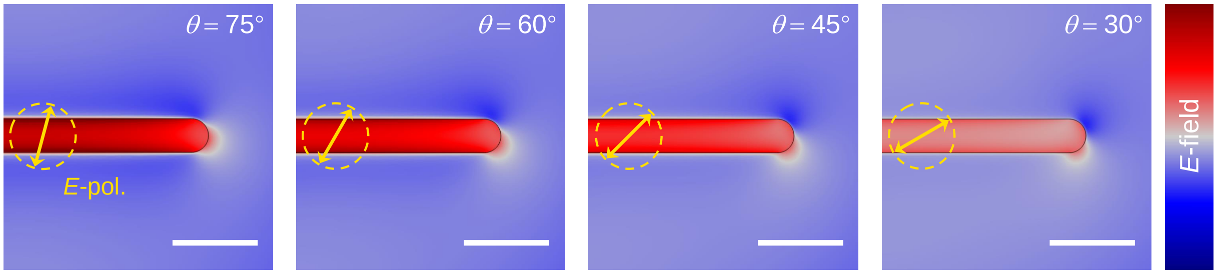

Fig. S4|Simulations of the polarization-controlled ablation front rotation. With the angle between laser polarization and groove (θ) decreasing (from left to right), the optical hot spot locating at the tip-end rotates accordingly. All scale bars are 50 nm.

5.3 Orientation of the near-field enhancement

The orientation of the optical hot spot can be rotated by changing the polarization of far-field beam (Fig. S4). As the angle between the polarization and the edge of nanogroove (θ) decreasing, the energy confined in the slot will also get smaller and smaller since only the E-field component perpendicular to the slot ($E_\perp = E\cos(\pi/2 - \theta)$) is enhanced. The vectorial near-field enhancement feature can be further confirmed by the experiment shown in Fig. S5. After initial seeding with pulse energy $\sim 10\%$ above the threshold, subsequent pulses with energy $\sim 20\%$ lower than threshold were utilized for raster scan (orange dash rectangle marked in Fig. S5). Here, the azimuth of E-field was modulated into a time-dependent sin-wave form. It's clear to see that the boundary of the ablated region (yellow dot-dash in Fig. S5) also evolved into a sin-wave shape (Supplementary Movie 1). This is also beneficial to the robustness of the direct writing since the ablation front is simultaneously determined by the structure and polarization (Fig. 3**a**).

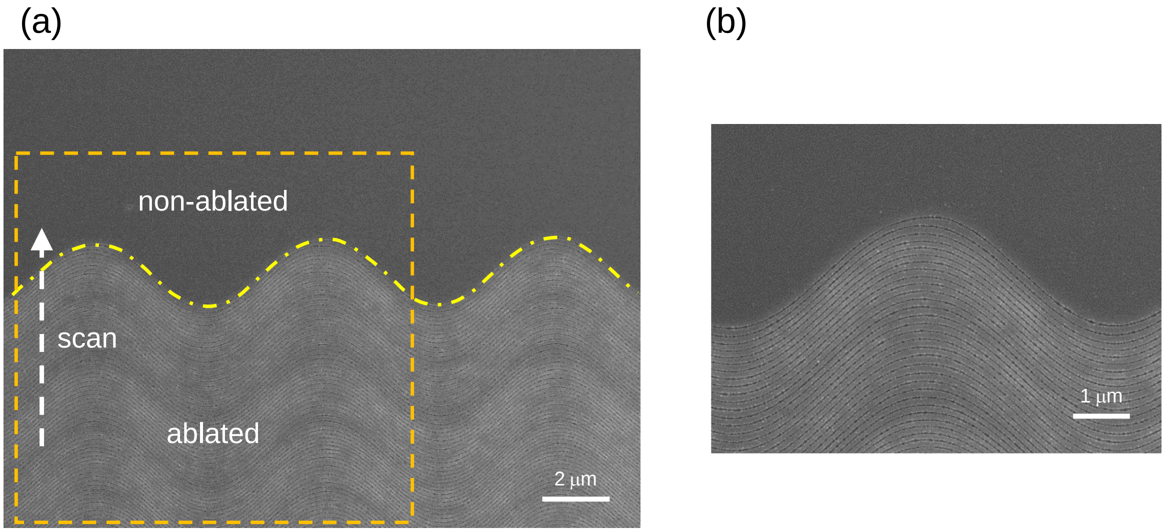

Fig. S5|Anisotropic patterning of O-FIB. (a) The ablated boundary evolved into a sin-wave shape following the time-dependent polarization azimuth, while the trajectory of the scanning beam is rectangular. (b) Close-up-view of ablated boundary.

Figure. S6 shows the angular dependence of area patterning at fixed polarization. As direction of scan v_s was closer to perpendicular in respect to the linear polarization of E-field of light, the pattern gradually acquired disorder. The best quality pattern were obtained for $\mathbf{v}_s \parallel \mathbf{E}$. The dipole nature of the scattered pattern is responsible for such writing principle. The boundary condition for E-field enhancement works for the direction normal to the edge of nanogroove ($\theta = \pi/2$). When polarization makes an angle $\theta < \pi/2$ with the edge of nanogroove, the light enhancement is weaker by a projection factor of $\cos^2(\pi/2 - \theta)$. In this case, light enhancement is not sufficient enough to sustain a much uniform extension, therefore the direction of v_s become significant to achieve uniform structures through light confinement effect.

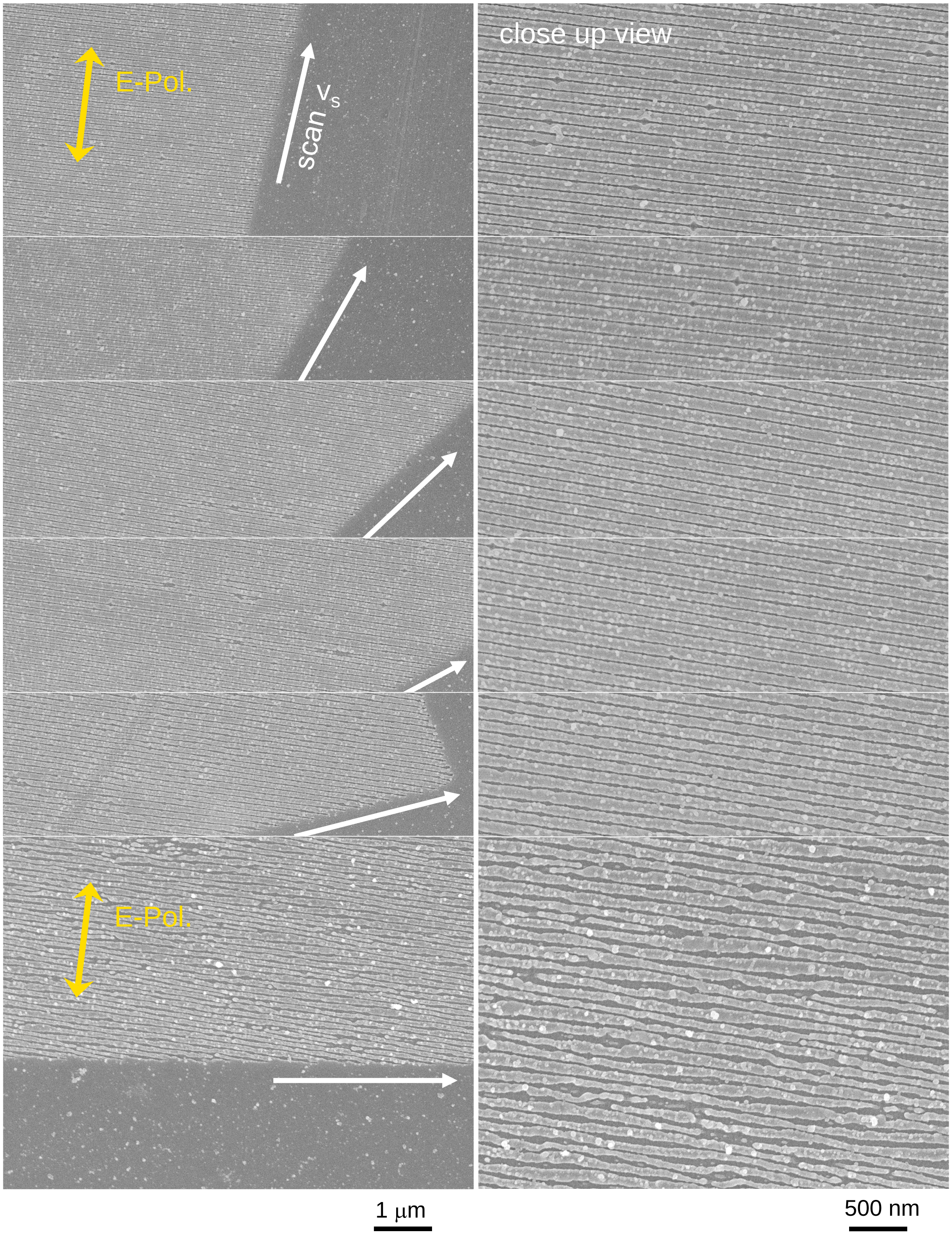

Fig. S6|Angular dependence of laser patterning by raster scanning. The scan speed was $v_s = 40$ μm/s at $f = 1$ kHz repetition rate, pulse energy $E_p = 17.2$ nJ at focus.

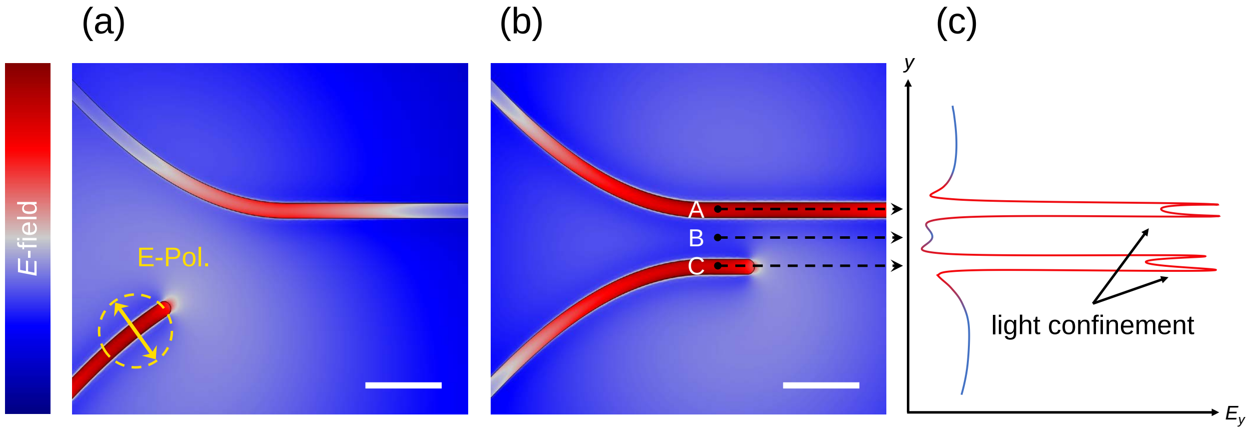

Fig. S7|Simulation of the electric field strength when two adjacent slots are approaching to each other at deep-subwavelength scale. (a) Separated nanogrooves. (b) Approached nanogrooves. (c) E-field strength profile along the cross section determined by point A and C. Point B indicates the minimum of E-field induced by the boundary condition, which plays a crucial role for the absence of the proximity effect.

5.4 Absence of the proximity effect

The evanescent nature of the localized near-field enhancement guides a merger of the nanogrooves down to deep-sub-wavelength level of $\lambda/10 \sim \lambda/16$ (Fig. S7). The near-field ablation is advancing while the neighbouring nanogroove (already existing) concentrates light. The region between two nanogrooves has exactly the minimum value of E-field (see point C in Fig. S7(b)). Absence of cross talk between adjacent slots is important for nano-writing applications since it eliminates the proximity effects (see Fig. 3**c** of the main text).

6 O-FIB at high irradiation: self-organization aspects

Nanogroove acts as light energy redistributor and, at an increasing pulse intensity, new satellite nanogrooves will be opened in a self-organized manner. Two secondary maxima of light intensity are formed on the (Fig. S8(a)) either side of the nanogroove parallel to the polarization as shown by light intensity distribution which is related to the electronic excitation of titania film. Numerically simulated separation of the two side lobes Λ was found closely following the experimental results and depended on the width D of the central groove (Fig. S8(b)). Two secondary nanogrooves were formed under a multi-pulse ($N > 20$) irradiation when the pulse energy was $\sim 10\%$ above the threshold of ablation. Intense scanning of the focal spot along direction of polarization with lateral displacement sets a pattern of quasi-periodic structures with period $\sim \Lambda/2$. This provides the theoretical foundation of the formation of seed array (Fig. 2**c** and Fig. 4**c** in the main text).

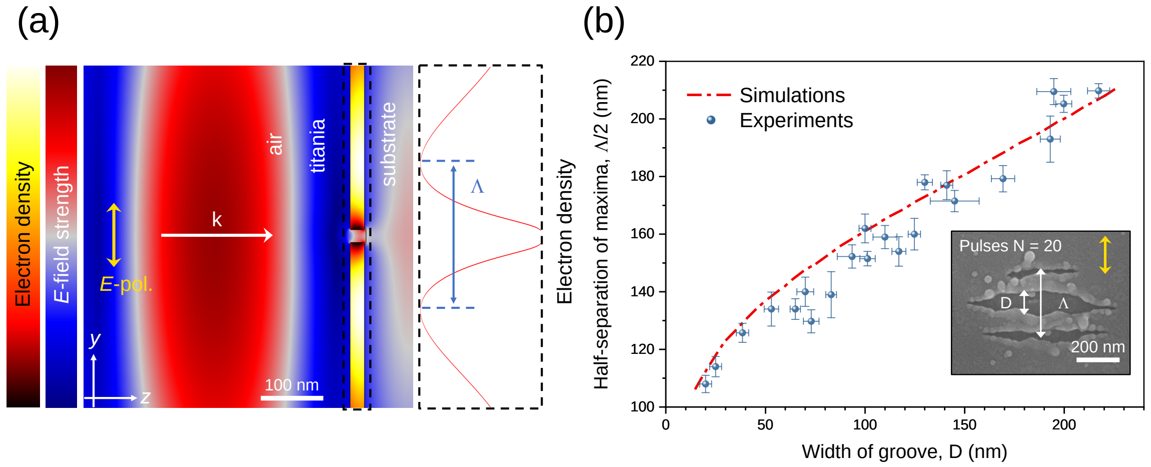

Fig. S8|Theoretical foundation of the formation of seed array. (a) Numerical 2D simulations of light intensity for a y-polarized plane wave illumination (along wavevector k) of 20-nm-wide nanogroove (along x-axis) in a 22.5-nm-thick titania film. Electron density profile was calculated for the experimentally implemented intensity. (b) Experimentally measured and numerically modeled dependence of the separation between two side maximum of light intensity vs. width of the central nanogroove $\Lambda \propto D$. Inset shows the SEM image with definitions of Λ, D.

7 References

1. Öktem, B. *et al.* Nonlinear laser lithography for indefinitely large-area nanostructuring with femtosecond pulses. *Nat. Photon.* **7**, 897–901 (2013).

2. Landau, L. *et al.* *Electrodynamics of continuous media*, vol. 8 (Elsevier, 2013).

3. Lax, M., Louisell, W. H. & McKnight, W. B. From Maxwell to paraxial wave optics. *Phys. Rev. A* **11**, 1365 (1975).

4. Richards, B. & Wolf, E. Electromagnetic diffraction in optical systems, II. Structure of the image field in an aplanatic system. *Proc. Royal Soc. London; Series A. Mathem. Phys. Sci.* **253**, 358–379 (1959).

5. Rethfeld, B. Unified model for the free-electron avalanche in laser-irradiated dielectrics. *Phys. Rev. Lett.* **92**, 187401 (2004).

6. Gamaly, E. *et al.* Modification of refractive index by a single femtosecond pulse confined inside a bulk of a photorefractive crystal. *Phys. Rev. B* **81**, 054113 (2010).

7. Malinauskas, M., Žukauskas, A., Bičkauskaitė, G., Gadonas, R. & Juodkazis, S. Mechanisms of three-dimensional structuring of photo-polymers by tightly focussed femtosecond laser pulses. *Opt. Express* **18**, 10209–10221 (2010).

8. Keldysh, L. Ionization in the field of a strong electromagnetic wave. *Sov. Phys. JETP* **20**, 1307–1314 (1965).

9. Buschlinger, R., Nolte, S. & Peschel, U. Self-organized pattern formation in laser-induced multiphoton ionization. *Phys. Rev. B* **89**, 184306 (2014).

10. Sokolowski-Tinten, K. & von der Linde, D. Generation of dense electron-hole plasmas in silicon. *Phys. Rev. B* **61**, 2643 (2000).

11. Rudenko, A., Colombier, J.-P. & Itina, T. E. From random inhomogeneities to periodic nanostructures induced in bulk silica by ultrashort laser. *Phys. Rev. B* **93**, 075427 (2016).

12. Hummel, R. E. Electrical properties of materials. In *Understanding Materials Science*, 180–216 (Springer, 1998).

13. Stuart, B. C. *et al.* Nanosecond-to-femtosecond laser-induced breakdown in dielectrics. *Phys. Rev. B* **53**, 1749 (1996).

14. Gildenburg, V. & Pavlichenko, I. High contrast periodic plasma pattern formation during the laser-induced breakdown in transparent dielectric. *Phys. Plasmas* **24**, 122306 (2017).

15. Kormann, C., Bahnemann, D. W. & Hoffmann, M. R. Preparation and characterization of quantum-size titanium dioxide. *J. Phys. Chem.* **92**, 5196–5201 (1988).

16. Enright, B. & Fitzmaurice, D. Spectroscopic determination of electron and hole effective masses in a nanocrystalline semiconductor film. *J. Phys. Chem.* **100**, 1027–1035 (1996).

17. Juodkazis, S., Rode, A. V., Gamaly, E. G., Matsuo, S. & Misawa, H. Recording and reading of three-dimensional optical memory in glasses. *Appl. Phys. B* **77**, 361–368 (2003).
